# Into the deep: the functionality of mesopelagic excursions by an oceanic apex predator

**DOI:** 10.1002/ece3.2260

**Published:** 2016-06-28

**Authors:** Lucy A. Howey, Emily R. Tolentino, Yannis P. Papastamatiou, Edward J. Brooks, Debra L. Abercrombie, Yuuki Y. Watanabe, Sean Williams, Annabelle Brooks, Demian D. Chapman, Lance K.B. Jordan

**Affiliations:** ^1^ Microwave Telemetry, Inc.ColumbiaMarylandUSA; ^2^ Department of Biological SciencesFlorida International UniversityNorth MiamiFloridaUSA; ^3^ Shark Research and Conservation ProgramCape Eleuthera InstituteEleutheraThe Bahamas; ^4^ Abercrombie & FishMiller PlaceNew YorkUSA; ^5^ National Institute of Polar ResearchTachikawaTokyoJapan; ^6^ Department of Polar ScienceSOKENDAI (The Graduate University for Advanced Studies)TachikawaTokyoJapan; ^7^ School of Marine and Atmospheric Science & Institute for Ocean Conservation ScienceStony Brook UniversityStony BrookNew YorkUSA

**Keywords:** Apex predator, deep‐diving behavior, foraging, k‐means cluster analysis, mesopelagic zone, satellite tagging

## Abstract

Comprehension of ecological processes in marine animals requires information regarding dynamic vertical habitat use. While many pelagic predators primarily associate with epipelagic waters, some species routinely dive beyond the deep scattering layer. Actuation for exploiting these aphotic habitats remains largely unknown. Recent telemetry data from oceanic whitetip sharks (*Carcharhinus longimanus*) in the Atlantic show a strong association with warm waters (>20°C) less than 200 m. Yet, individuals regularly exhibit excursions into the meso‐ and bathypelagic zone. In order to examine deep‐diving behavior in oceanic whitetip sharks, we physically recovered 16 pop‐up satellite archival tags and analyzed the high‐resolution depth and temperature data. Diving behavior was evaluated in the context of plausible functional behavior hypotheses including interactive behaviors, energy conservation, thermoregulation, navigation, and foraging. Mesopelagic excursions (*n *=* *610) occurred throughout the entire migratory circuit in all individuals, with no indication of site specificity. Six depth‐versus‐time descent and ascent profiles were identified. Descent profile shapes showed little association with examined environmental variables. Contrastingly, ascent profile shapes were related to environmental factors and appear to represent unique behavioral responses to abiotic conditions present at the dive apex. However, environmental conditions may not be the sole factors influencing ascents, as ascent mode may be linked to intentional behaviors. While dive functionality remains unconfirmed, our study suggests that mesopelagic excursions relate to active foraging behavior or navigation. Dive timing, prey constituents, and dive shape support foraging as the most viable hypothesis for mesopelagic excursions, indicating that the oceanic whitetip shark may regularly survey extreme environments (deep depths, low temperatures) as a foraging strategy. At the apex of these deep‐water excursions, sharks exhibit a variable behavioral response, perhaps, indicating the presence or absence of prey.

## Introduction

Telemetry data have revealed a number of pelagic fishes take deep‐water excursions to meso‐ and bathypelagic depths (>200 m), representing a link and possibly transferring nutrients among vertical strata of the water column (Sutton 2013; Thorrold et al. 2014). In air‐breathing marine animals, diving behavior is assumed to be an exclusive tradeoff between oxygen recovery at the surface and time spent at depth (Dunphy‐Daly et al. 2010). However, in ectothermic animals which acquire oxygen through gas exchange via gills, the motives are more ambiguous. The functional importance of deep diving (below the deep scattering layer [DSL]) in pelagic fishes varies among species and is likely attributed to one or more of the following categories: interactive behaviors, energy conservation, behavioral thermoregulation, navigation, and foraging (Carey and Scharold 1990; Graham et al. 2006; Schaefer et al. 2007; Weng et al. 2007; Gore et al. 2008; Brunnschweiler et al. 2009; Skomal et al. 2009; Willis et al. 2009; Wilson and Block 2009; Campana et al. 2011; Gleiss et al. 2011; Saunders et al. 2011; Howey‐Jordan et al. 2013; Braun et al. 2014; Holmes et al. 2014; Vaudo et al. 2014; Phillips et al. 2015). Blue sharks (*Prionace glauca*), scalloped hammerhead sharks (*Sphyrna lewini*), and yellowfin tunas (*Thunnus albacares*) are suspected to dive to forage on concentrations of DSL organisms (Carey and Scharold 1990; Schaefer et al. 2007; Hoffmayer et al. 2013). Deep dives demonstrated by white sharks (*Carcharodon carcharias*) are hypothesized to serve the purpose of either foraging or reproduction (Jorgensen et al. 2012). Other studies indicate consistent and constant vertical movement (i.e., oscillatory diving), as demonstrated by scalloped hammerheads and southern bluefin tunas (*Thunnus maccoyii*), may represent navigational referencing as deep diving allows access to geomagnetic and bathymetric cues (Klimley 1993; Willis et al. 2009). Furthermore, diving behavior in whale sharks (*Rhincodon typus*) and reef manta rays (*Manta alfredi*) may be attributed to energy conservation (Gleiss et al. 2011; Braun et al. 2014), as dive geometry may vary based on optimization of vertical or horizontal search patterns (Gleiss et al. 2011). The diversity of unverified hypotheses highlights the need for further study to elucidate the functionality of deep‐diving behavior in fishes that generally associate with epipelagic waters.

The tolerance of epipelagic fishes to environmental conditions encountered during meso‐ and bathypelagic excursions may be limiting (Jorgensen et al. 2009; Wilson and Block 2009). In ectothermic animals, temperature is an important abiotic factor; as physiologists have long asserted that thermal habitat use and thermal physiology are closely coadapted, such that temperature use maximizes fitness (Martin and Huey 2008). Similarly, oxygen concentration has been observed to limit depth range in sharks (Nasby‐Lucas et al. 2009); expanding pelagic hypoxic regions may reduce available habitat and prey resources (Gilly et al. 2013). As such, there remains a need to understand the way animals react to environmental variables and use different habitats.

High‐resolution data are required to assess such hypotheses. Biologging technology is commonly used to collect sequential point measurements in order to study vertical habitat use in fishes (Gleiss et al. 2009; Whitney et al. 2010; Nakamura et al. 2011). While providing high‐resolution data and an effective means to reconstruct time‐series depth and temperature profiles, the need for physical recovery or active tracking complicates these methodologies. Pop‐up satellite archival tags (PSATs) provide a solution by collecting data in situ with subsequent transmission through a constellation of satellites. This method is also not without limitation, as tag battery life and data throughput constraints dictate the data resolution and quantity received. However, higher resolution data are stored within the archival memory so that, in the event of physical tag recovery, the complete archived dataset can be retrieved.

Here, we use an unusually large time‐paired depth and temperature dataset derived from physically recovered PSATs to assess hypotheses regarding the functionality of deep (>200 m) diving in oceanic whitetip sharks (*Carcharhinus longimanus*). A previous study using PSATs showed that adult female oceanic whitetip sharks spent most of their tracked time in the upper 100 m of the water column (Howey‐Jordan et al. 2013). However, like many epipelagic fishes, oceanic whitetip sharks make occasional deep dives into (and beyond) the mesopelagic zone, tentatively hypothesized to represent foraging behavior (Howey‐Jordan et al. 2013). To gain insight into the function, execution, and possible motives of these mesopelagic excursions in a pelagic shark, we investigated spatial and temporal distribution of deep (>200 m) dives, identified dive profile shapes, and examined causative factors for isolated dive events. Results were assessed within the context of plausible hypotheses suggested as functional explanations for deep‐diving behavior in epipelagic fishes. The resulting empirical model was designed to be applied to other marine vertebrates.

## Methods

Standard Rate (SR) X‐Tags (Microwave Telemetry, Inc., Columbia, MD) were deployed on oceanic whitetip sharks during May 2011–2013 near Cat Island, The Bahamas (24.12°N, 75.28°W) (Table S1) (see Howey‐Jordan et al. 2013). Many tags washed ashore during the transmission phase, and use of real‐time Argos locations directed recovery efforts. Although X‐Tags transmit a subset of time‐series data through the Argos system, physical recovery of the tag allows for extraction of the entire high‐resolution 2‐min archived dataset. Recovered SR X‐Tags provide daily light‐based geolocation estimates and records with 2‐min sampling rate for depth (0.34 m resolution), temperature (0.16–0.23°C resolution), and light level (http://www.microwavetelemetry.com/fish). Prior to analysis, the 2‐min sampling interval was assessed for adequacy in capturing oceanic whitetip vertical movements by comparison with high‐resolution depth data (1 Hz, ±0.25 m resolution) obtained from an individual tagged with a PD3GT data logger (Little Leonardo, Tokyo, Japan) (Watanabe et al. 2015) (Data S1).

### Data treatment

A mesopelagic excursion (ME) was characterized as a sequence of ≥5 consecutive depth records below the 200 m isobath, consistent with the boundary between the epipelagic and mesopelagic zones. The start of the ME was represented by the last depth record above 200 m, and the end of the ME was identified as the first depth record above 200 m. Therefore, each ME contained ≥7 depth records. Any dive that did not meet these criteria was omitted from analysis. ME depth‐versus‐time profiles were annotated with dissolved oxygen concentration (Garcia et al. 2013) and tag‐recorded temperature. Additionally, each ME event was assigned an approximate location, diel period (dawn, day, dusk, or night), and lunar phase (first quarter, full moon, last quarter, and new moon) (Data S2). Daily sea surface temperature (SST) estimates were based on daily maximum temperature records (Galuardi and Lutcavage 2012). The oxygen minimum zone (OMZ) was defined as the region with oxygen values ≤3.5 mL/L (Stramma et al. 2012).

### Statistical analysis

We used R 2.15.3 for all computations (R Core Team 2013) and specified a significance level of 0.05 for all analyses. Pearson's product moment (*r*) and Spearman's rank (*r*_s_) were used for correlations.

#### Spatial distribution

Using the methods of McAdam et al. (2012), daily filtered light‐level location estimates (see Data S2) corresponding to days including an ME were compared to the locations where no MEs were recorded, while accounting for repeated observations from each individual. Additionally, ME locations were overlaid on the 25%, 75%, and 100% utilization distribution contours calculated from the filtered positions of all individuals (Galuardi 2012; Galuardi and Lutcavage 2012).

#### Cluster analysis

Depth‐versus‐time profiles of MEs were separated into descent and ascent phases. Descent profiles included all depth records from the beginning of the dive down to and including the maximum depth record, and ascent profiles included the maximum depth record and all the subsequent dive records (Howey‐Jordan et al. 2013). K‐means clustering with Euclidean distance measure (Das et al. 1998; Schreer et al. 1998) was applied separately to standardized (depth and time) ascents and descents (Schreer and Testa 1995; Schreer et al. 1998). The clustering procedure was implemented nine times for selection of 2–10 clusters, and the appropriate number of clusters was determined as the point at which no redundant clusters were evident (Schreer et al. 1998). Additionally, *R* ^2^ values were considered with respect to the number of clusters (Schreer and Testa 1995, 1996; Schreer et al. 1998).

#### Transition point ascent analysis

One ascent cluster exhibited a noticeable shift in vertical velocity between two approximately constant vertical velocity (linear) segments. Broken‐stick regression (Toms and Lesperance 2003) was applied, with the “bentcableAR” R package (Chiu 2012), to each nonstandardized depth‐versus‐time profile in this cluster to estimate the time and depth at which the shift in vertical velocity occurred as well as estimate mean vertical velocity on each segment of the ascent. The transition point, marking the start of the second linear segment, was defined as the first dive record immediately after the model's real‐valued change‐point estimate. Any ascent that failed to obtain a transition point estimate due to limited number of records was omitted from the transition point analysis.

#### Generalized linear mixed models

Generalized linear mixed models (GLMMs) were developed to investigate various ME relationships, while accounting for the random effect due to individual shark. Models were constructed with the “lme4” (Bates et al. 2013) and “nlme” (Pinheiro et al. 2013) R packages, and simultaneous tests for general linear hypotheses with Tukey's contrasts were employed post hoc with the “glht” function from the “multcomp” package in R (Hothorn et al. 2008, 2013).

Mesopelagic excursion frequency was investigated with three negative binomial GLMMs, each containing a log‐transformed temporal offset term to account for dive rate (McCullagh and Nelder 1989; Zuur et al. 2009). Significance of model terms was assessed with an analysis of deviance test, comparing the null model to the model including the fixed‐effect factor in question (Zuur et al. 2009). First, ME frequency in relation to SST was examined by considering monthly dive frequency and monthly mean SST for each individual. In the remaining two models, we independently considered ME frequency with respect to the diel period factor and lunar phase factor. In each case, MEs were counted for each individual in the respective categories (Data S3, Table S2).

A linear mixed‐effects (LME) model was constructed to compare temperatures experienced prior to MEs to temperatures experienced during periods of time spent strictly in the epipelagic zone. Non‐ME mean temperatures were based on 50 random, 5‐h samples lacking MEs (nor within 1 hour of an ME) for each individual, and pre‐ME mean temperatures were based on the 5‐h period prior to each ME. Multiple time periods, besides the 5‐h samples, were considered (30 min, 1–4 h) (Data S3, Table S3).

Additionally, LME models were used to evaluate the dependence of ME characteristics on the ascent and descent cluster identifications. Six Box–Cox transformed response variables were considered: dive maximum depth (m), dive minimum temperature (°C), mean descent vertical velocity (m/s), mean ascent vertical velocity (m/s), dive minimum dissolved oxygen level (mL/L), and duration of the dive phase (minutes). Models for the first five response variables treated the ascent cluster factor and descent cluster factor as additive fixed effects, each containing three levels. The last model for the phase duration response variable used cluster identification as the single fixed effect containing six levels (Data S3, Table S3).

Lastly, Box–Cox transformed mean vertical velocity of the linear ascent and two segments of the transition point ascent were selected for comparison with an LME model. Mean vertical velocity was estimated by linear and broken‐stick regression (described above) for the linear ascent and transition point ascent, respectively (Data S3, Table S3).

## Results

### Sampling rate

The accelerometer package was deployed for 3.7 days on a mature female (FL = 216 cm), obtaining 315,555 depth records. Based on comparison between the 1‐sec profile (accelerometer) and the 2‐min subsample (representative of X‐Tag sampling rate), vertical movement of the oceanic whitetip was captured by the 2‐min sampling rate (Data S1, Fig. S1).

### Mesopelagic excursions

Sixteen recovered X‐Tags, retrieved from 14 females and 2 males, obtained 4,248,306 depth and temperature records from 2966 total tracking days, averaging 185.38 ± 90.61 (mean ± standard deviation) tracking days per individual (Table S1). Individuals spent the majority of tracked time (90.77 ± 6.76%) in the upper 100 m of the water column. Time spent below the epipelagic zone (>200 m) accounted for 0.34 ± 0.23% of recorded time. Of the 1555 dives below 200 m, 610 (39.2%) met the criteria of a mesopelagic excursion (ME) for analysis. All but one individual (female 129933) demonstrated MEs; this individual was tracked for only 22 days.

Maximum depths of MEs ranged from 202.1 to 1190.2 m with minimum temperatures ranging from 6.79 to 21.87°C. Most MEs did not encounter the OMZ (*n *=* *487, 79.8%). Other MEs registered maximum depth within the OMZ (*n *=* *103, 16.9%), and the remaining dives passed through the OMZ, into oxygen‐rich waters, before ascending (*n *=* *20, 3.3%). Duration of MEs averaged 21.01 ± 8.42 min (range: 11.95–64.12 min). In general, MEs were characterized by fast descents (6.61 ± 3.53 min) followed by significantly longer ascents (14.40 ± 7.16 min) (*F*_1,1204_ = 847.953, *P *<* *0.0001). A significant correlation between tag deployment duration and the total number of MEs was detected (*r* ^2* *^= 0.308, *P *=* *0.02563, *N *=* *16). No significant correlation was identified between animal size (FL) and scaled ME count (i.e., number of dives/day) (*r* ^2^ = 0.147, *P *=* *0.1431, *N *=* *16).

### Mesopelagic excursions and environmental variables

**Figure 1:**
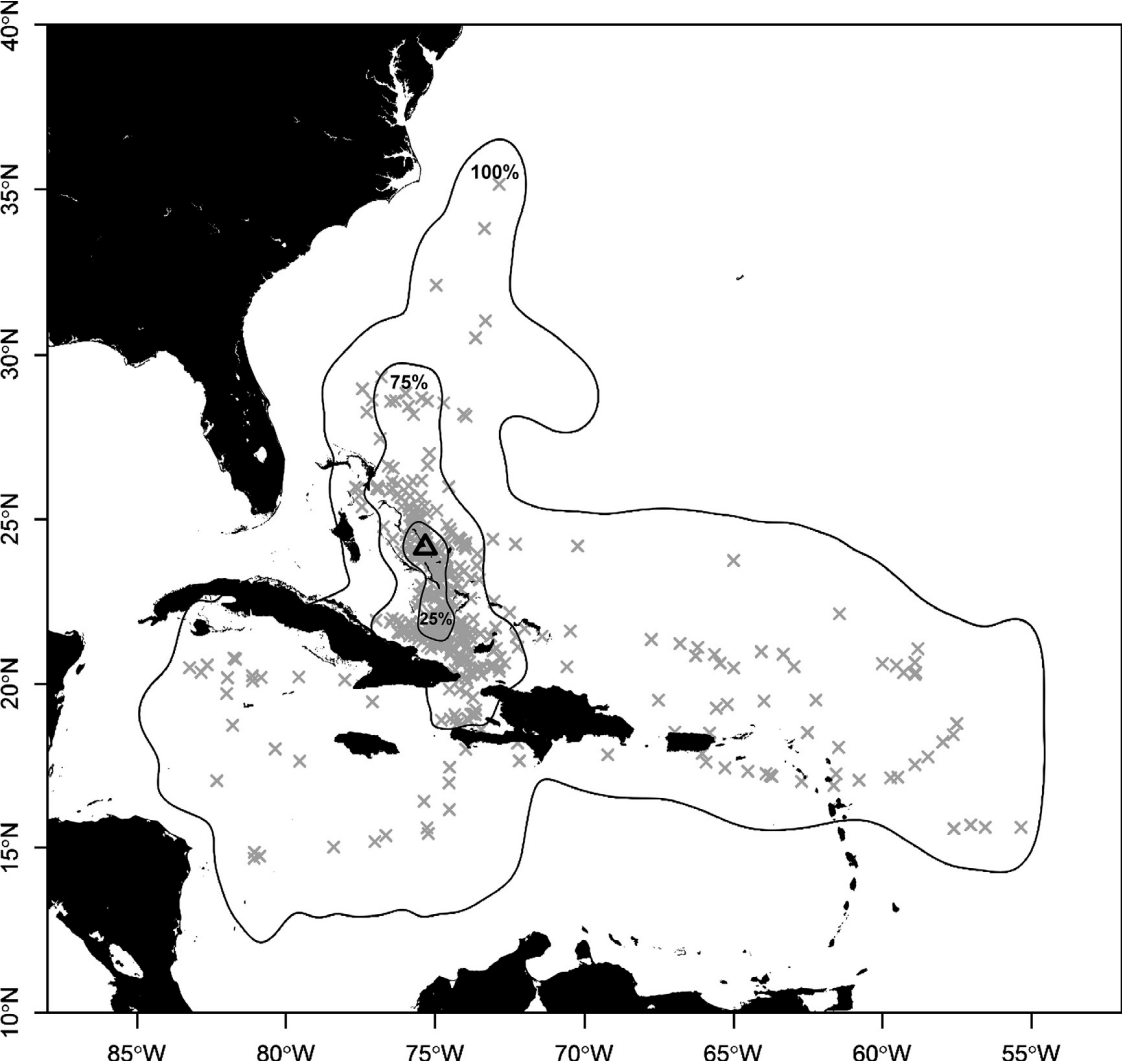
Map displaying 25%, 75%, and 100% utilization distribution contours from 16 recovered X‐Tags. Approximate mesopelagic excursion locations indicated by gray X and deployment location indicated by triangle.

No significant difference between the spatial distribution of locations with MEs and the distribution of locations without MEs was identified (*Ψ * =  1.3, *P *=* *0.865). Based on visual inspection, the spatial distribution of the MEs appeared to be randomly distributed within the 25%, 75%, and 100% utilization distribution contours (Fig. 1). MEs occurred in every month, with individual monthly ME counts ranging between 0 to 89 dives (5.7 ± 9.8 dives per month). Mean monthly SST made a significant contribution to the prediction of monthly ME frequency (negative binomial GLMM: $\chi_{1}^{2}$ = 23.41, *P *= 1.311 × 10^−6^). Specifically, a mean SST of 24°C predicted 0.93 MEs per month, varying between individuals (0.20–2.69 dives per month). For a warmer mean SST of 30°C, the model predicted 9.63 MEs per month, ranging greatly across individuals (1.28–38.81 dives per month).

Diel period was a significant predictor of ME frequency (negative binomial GLMM: $\chi_{3}^{2}$ = 35.95, *P *=* *7.672 × 10^−8^). The post hoc general linear hypotheses revealed the dusk period, when scaled by duration, contained significantly more MEs than the other three periods. The lunar phase factor was only a marginally significant predictor for the frequency of MEs (negative binomial GLMM: $\chi_{3}^{2}$ = 8.824, *P *=* *0.03172). General linear hypotheses revealed that the first quarter contained significantly more MEs than the new moon phase; however, we observed no convincing pattern indicative of a biologically significant lunar phase effect.

### Pre‐ME temperature analysis

The 5‐h period prior to MEs (26.08 ± 1.13°C) was significantly cooler than randomly selected 5‐h periods that were not associated with any ME (26.30 ± 0.94°C) (*t*_1344_ = −2.395, *P *=* *0.0167). Of the other time periods considered, the pre‐ME temperature was always cooler than the randomly selected periods strictly spent in the epipelagic zone; however, the relationship was only statistically significant in the models considering intervals of 3 and 5 hours.

### Dive phase profile identification

**Figure 2:**
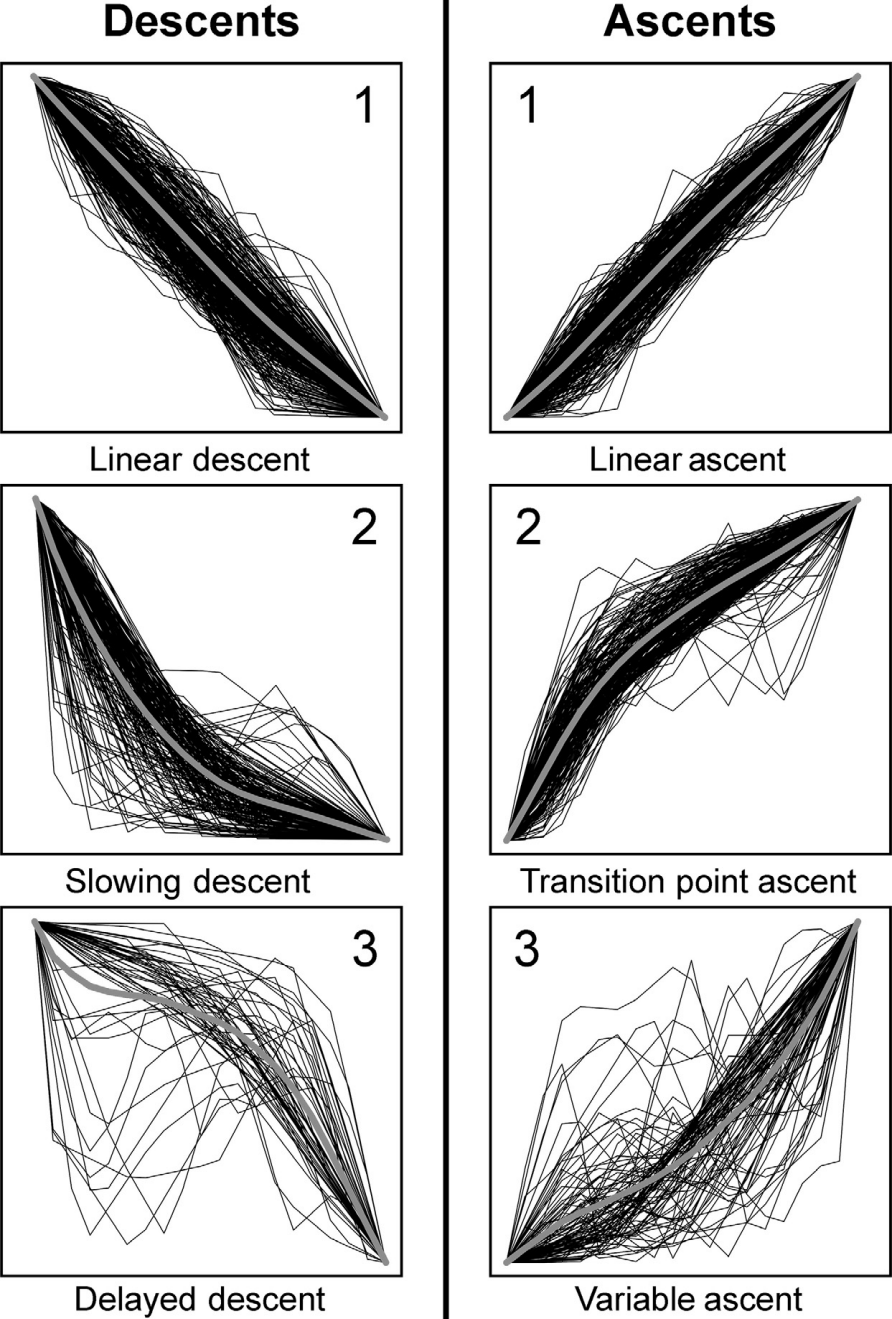
Standardized depth‐versus‐time descent and ascent profiles for each cluster. Thick gray curve represents mean profile for each cluster.

Cluster analysis identified three descent clusters (Descents 1–3) and three ascent clusters (Ascents 1–3) (Fig. 2). The selection of three clusters explained 66.51% and 67.55% of the ME profile shape variation for descents and ascents, respectively. The first descent cluster, Descent 1, contained the majority of descents (Table 1) and was characterized by an approximately linear profile (corresponding to constant vertical velocity) (Fig. 2). Descent 2 was considered the slowing descent group as vertical velocity decreased as profiles approached maximum depth (Fig. 2). Descent 3 cluster contained the fewest descents (Table 1). Although this group exhibited erratic changes in vertical velocity (and often vertical direction), vertical velocity generally increased when approaching maximum depth, classifying this cluster as the delayed descent group (Fig. 2).

**Table 1:** Pairings between mesopelagic excursion (ME) ascent and descent clusters represented as occurrences and corresponding percentages of the entire ME dataset

ME Phase cluster	Descent 1	Descent 2	Descent 3	Total occurrences
Ascent 1	*n *=* *149 24.43%	*n *=* *116 19.02%	*n *=* *24 3.93%	289
Ascent 2	*n *=* *152 24.92%	*n *=* *51 8.36%	*n *=* *24 3.93%	227
Ascent 3	*n *=* *55 9.02%	*n *=* *35 5.74%	*n *=* *4 0.66%	94
Total occurrences	356	202	52	610

The Ascent 1 cluster represented the most common ascent profile (Table 1). This cluster was approximately linear indicating near‐constant vertical velocity and was therefore termed the linear ascent (LA) group (Fig. 2). The second most common cluster, Ascent 2, identified as the transition point ascent (TPA) group was characterized by an abrupt decrease in vertical velocity after a comparatively fast initial ascent period commencing at the dive apex. Before and after the transition point, the ascent was approximately linear for the majority of profiles (Fig. 2). The remaining MEs were categorized in the Ascent 3, variable ascent cluster and exhibited random vertical variations in velocity and direction (Fig. 2).

Pairing corresponding descent and ascent phases (to represent a complete ME profile) revealed that nearly 50% of MEs were comprised of Descent 1 with either Ascent 1 or Ascent 2 (Table 1). All deep‐diving individuals (*n *=* *15) exhibited MEs classified in these three clusters.

### Dive phase characteristics

Linear mixed‐effect models revealed relationships between ME phase shapes and dive characteristics. Descent shape was a significant predictor for only one of the five dive characteristics examined (Table 2). Mean descent vertical velocity significantly differed between the three descent shapes (*F*_2,591_ = 19.68088, *P *<* *0.0001). Descent 1 was the fastest group, while Descent 3 was the slowest (Table 2). Descent shape was a poor predictor for dive maximum depth (*F*_2,591_ = 0.5, *P *=* *0.6001), dive minimum temperature (*F*_2,591_ = 0.1147, *P *=* *0.8917), mean vertical velocity along the subsequent ascent (*F*_2,591 _= 2.3894, *P *=* *0.0926), and minimum dissolved oxygen concentration (*F*_2,591 _= 2.5864, *P *=* *0.0761) (Table 2).

**Table 2:** Mean ± SD and range provided for each dive variable in each dive phase cluster

	Descent 1	Descent 2	Descent 3	Ascent 1	Ascent 2	Ascent 3
Dive maximum	452.7 ± 199.0	393.0 ± 139.7	494.6 ± 246.9	372.3 ± 113.7^b^	584.1 ± 200.5^a^	277.6 ± 67.28^c^
depth (m)	202.1–1190.2	208.1–828.4	204.1–1081.9	205.8–751.8	204.1–1190.2	202.1–482.5
Dive minimum	16.60 ± 3.40	17.43 ± 2.58	16.01 ± 3.84	17.95 ± 1.90^b^	14.36 ± 3.54^c^	19.32 ± 1.22^a^
temperature (°C)	6.79–21.87	9.48–21.20	7.75–21.37	10.89–21.37	6.79–20.70	14.78–21.87
Mean descent	1.00 ± 0.68^a^	0.71 ± 0.41^b^	0.55 ± 0.39^c^	0.75 ± 0.55^b^	1.19 ± 0.59^a^	0.43 ± 0.38^c^
vertical velocity (m/s)	0.045–2.898	0.043–1.754	0.006–1.613	0.022–2.624	0.006–2.898	0.042–2.164
Mean ascent	0.31 ± 0.18	0.26 ± 0.16	0.30 ± 0.17	0.26 ± 0.14^b^	0.39 ± 0.16^a^	0.13 ± 0.10^c^
vertical velocity (m/s)	0.003–0.941	0.012–0.850	0.015–0.980	0.024–0.980	0.009–0.941	0.003–0.489
Dive minimum	3.94 ± 0.44	4.04 ± 0.38	3.74 ± 0.48	4.10 ± 0.33^b^	3.67 ± 0.43^c^	4.22 ± 0.26^a^
dissolved oxygen (mL/L)	2.83–4.66	2.98–4.63	2.92–4.49	2.83–4.62	2.85–4.52	3.19–4.66
Duration (min)	5.84 ± 3.01^e^ 1.97–22.02	6.92 ± 2.77^d^ 3.98–18.05	10.70 ± 5.83^c^ 3.98–32.00	12.62 ± 6.87^b^ 2–56.1	17.30 ± 6.97^a^ 6–51.95	12.83 ± 6.22^b^ 4–36.02

Differing lowercase letters indicate significant differences between clusters determined from general linear hypotheses post hoc test with Tukey's contrasts. First five rows compare descent and ascent clusters independently, while row six (Duration) compares all six clusters.

In contrast, ascent shape was a significant predictor for all of the dive characteristics considered (Table 2). Dive maximum depth and minimum temperature significantly differed between ascent shapes (maximum depth: *F*_2,591 _= 202.2, *P *<* *0.0001; minimum temperature: *F*_2,591 _= 171.3835, *P *<* *0.0001). Ascent 2 corresponded to the deepest and coldest dives, whereas Ascent 3 corresponded to the shallowest and warmest dives (Table 2). Ascent shape associated with the mean vertical velocity of the preceding (corresponding) descent (*F*_2,591 _= 88.1210, *P *<* *0.0001). Ascent 2 was preceded by the fastest descents, and Ascent 3 followed the slowest descents (Table 2). Similarly, ascent shape was significantly associated with mean vertical velocity of the ascent (*F*_2,591 _= 132.0856, *P *<* *0.0001). Ascent 2 was the fastest group followed by Ascent 1, and Ascent 3 was the slowest group (Table 2). Lastly, ascent cluster significantly predicted the minimum dissolved oxygen experienced during the dive (*F*_2,591 _= 109.9013, *P *<* *0.0001). Dives in the Ascent 2 group encountered the lowest oxygen levels, while dives in the Ascent 3 group recorded the highest minimum oxygen levels (Table 2). Although differences were identified between ascent phase groups, there was still considerable overlap of the profiles in the water column (Table 2).

Cluster designation was a significant predictor for dive phase duration (*F*_5,1200 _= 240.22, *P *<* *0.0001). All descent clusters were significantly shorter in duration than all ascent clusters. Ascent 2 was the longest in duration, and Descent 1 was the shortest in duration. All clusters significantly differed from each other in terms of duration except for Ascent 1 and Ascent 3 (Table 2).

### Transition point ascent

**Figure 3:**
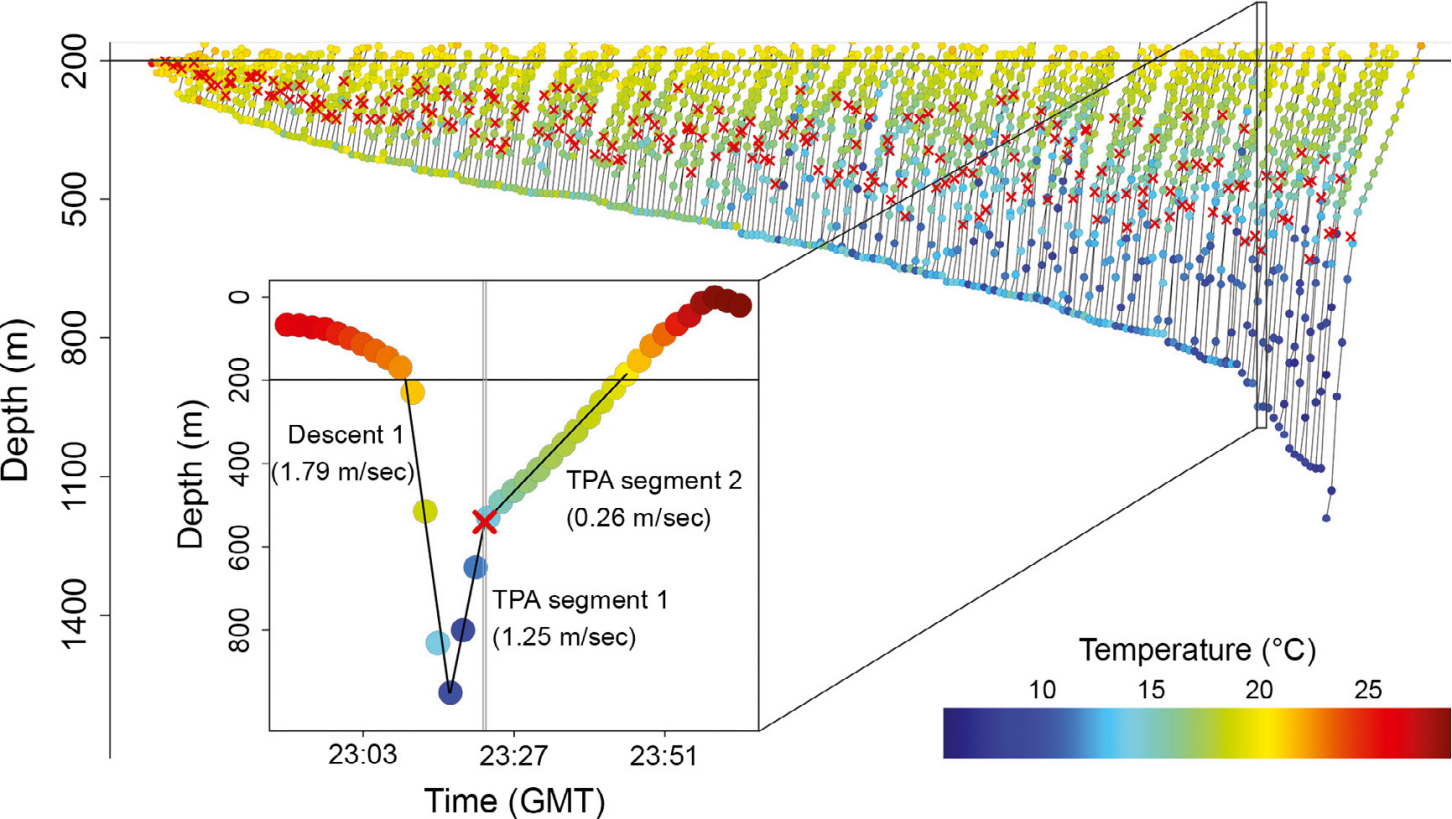
Concatenated depth‐versus‐time transition point ascents (*n *=* *224) displayed in the order of maximum dive apex depth and colored by temperature. Red X indicates change‐point estimated by broken‐stick regression. Inset: complete mesopelagic excursion from recovered X‐Tag 107797, including nine additional records above 200 m before and after the dive event. Dive profile has linear descent (Descent 1) followed by a transition point ascent (Ascent 2). Solid black lines overlaid on the descent and ascent represent linear and broken‐stick regression model fits, respectively, and corresponding vertical velocity (linear slopes) are indicated. Change‐point confidence intervals (95%) indicated by gray vertical lines.

Transition points were calculated with broken‐stick regression for 224 profiles (98.7%) in the Ascent 2 (i.e., transition point ascent [TPA]) cluster (Fig. 3). The initial ascent before the transition point (labeled TPA Segment 1 in Fig. 3 inset) lasted for 6.01 ± 2.66 min, and the final ascent after the transition point (labeled TPA Segment 2 in Fig. 3 inset) lasted 11.45 ± 5.91 min. Mean change in vertical velocity at the transition point was −0.53 ± 0.31 m/s, indicating a decrease in vertical velocity at the transition point.

### Ascent vertical velocity comparison

In the comparison between Ascent 1 and the two segments of Ascent 2 (i.e., TPA Segment 1 and TPA Segment 2), cluster (or cluster segment) designation was a significant predictor for mean vertical velocity magnitude (*F*_2,720 _= 348.4038, *P *<* *0.0001). No difference was identified between the vertical velocity of Ascent 1 (0.26 ± 0.15 m/s) and the second segment (Segment 2) of Ascent 2 (0.26 ± 0.10 m/s). The two segments of Ascent 2 significantly differed from each other; the initial vertical velocity (TPA Segment 1) was significantly faster than the vertical velocity after the transition point (TPA Segment 2).

### Ascent shape and environmental variables

Mean vertical velocity of Ascent 1 and two segments of Ascent 2 significantly correlated with dive maximum depth, minimum temperature, and minimum oxygen (Table 3). Independent of ascent phase, mean vertical velocity along the ascent increased as dives encountered more extreme environments (i.e., colder, deeper, and lower oxygen). However, of the ascent phases considered, Ascent 2 Segment 1 exhibited the strongest relationships with the considered variables, whereas Ascent 2 Segment 2 demonstrated the weakest relationships. Furthermore, across all ascent types, vertical velocity demonstrated the weakest relationship with dive minimum oxygen when compared to minimum temperature and maximum depth (Table 3).

**Table 3:** Spearman's rank correlations and corresponding *P* values for relationships between vertical velocity of Ascent 1 (linear ascent) and Ascent 2 (transition point ascent) with respect to environmental variables

Ascent phase vertical velocity	Dive maximum depth	Dive minimum temperature	Dive minimum oxygen
Ascent 1 (*n *=* *289)	*r*_s_ = 0.6516 *P *<* *2.2 × 10^−16^	*r*_s_ = −0.5868 *P *<* *2.2 × 10^−16^	*r*_s_ = −0.1858 *P *=* *0.001515
Ascent 2 Segment 1 (*n *=* *224)	*r*_s_ = 0.7658 *P *<* *2.2 × 10^−16^	*r*_s_ = −0.7064 *P *<* *2.2 × 10^−16^	*r*_s_ = −0.6125 *P *<* *2.2 × 10^−16^
Ascent 2 Segment 2 (*n *=* *224)	*r*_s_ * *= 0.4318 *P *<* *1.38 × 10^−11^	*r*_s_ * *= −0.3134 *P *<* *1.70 × 10^−6^	*r*_s_ * *= −0.1895 *P *=* *0.0044

Considering only Ascent 2, transition point depth positively correlated with dive maximum depth (*r*_*s*_ = 0.8078, *P *<* *2.2 × 10^−16^, *N *=* *224); deeper dives associated with deeper transition points (Fig. 3). In addition, the depth range of Segment 1 positively correlated with dive maximum depth (*r*_s_ = 0.9053, *P *<* *2.2 × 10^−16^, *N *=* *224) such that deeper dives exhibited a greater Segment 1 depth range. Similarly, transition point temperature correlated with dive minimum temperature (*r*_s_ = 0.8969, *P *<* *2.2 × 10^−16^, *N *=* *224), indicating that colder dives had colder transition points (Fig. 3). Furthermore, Segment 1 temperature range correlated with dive minimum temperature (*r*_s_ = −0.8999, *P *<* *2.2 × 10^−16^, *N *=* *224), indicating that dives to colder environments experienced a greater change in temperature between the dive apex and transition point. The relationship between the transition point oxygen concentration and dive apex oxygen concentration was also correlated (*r*_s_ = 0.5551, *P *<* *2.2 × 10^−16^, *N *=* *224).

## Discussion

### Description of mesopelagic excursions

Physically recovered X‐Tags allowed for extraction of archival data, producing an amount of depth and temperature records unprecedented in other shark tagging studies. Although oceanic whitetip sharks predominately associate with epipelagic waters, the high‐resolution time‐series data revealed deep diving events (termed mesopelagic excursions [MEs]), into meso‐ and bathypelagic zones, demonstrated by all individuals instrumented for longer than 22 days. The analysis comparing 1‐sec (accelerometer) and 2‐min sampling rates confirmed that recovered X‐Tags proved sufficient for capturing oceanic whitetip vertical behavior (Data S1, Fig. S1). MEs occurred year round during migrations and within The Bahamas, near the aggregation site. ME frequency exhibited a significant relationship with monthly mean SST, diel period, and lunar phase. However, the highly variable relationships with SST or lunar phase suggest these factors were unlikely catalysts for MEs.

Nearly all oceanic whitetip MEs lacked a detectable bottom phase, as seen in other deep‐diving species (e.g., whale sharks, yellowfin tunas, Chilean devil rays [*Mobula tarapacana*], basking sharks [*Cetorhinus maximus*]), and were generally represented by a monotonic descent to the dive apex followed by a monotonic ascent (Schaefer et al. 2007; Gore et al. 2008; Thums et al. 2012; Thorrold et al. 2014). Cluster analysis of oceanic whitetip MEs revealed three unique descent shapes and three unique ascent shapes. However, the shape associated with slowing descents (Descent 2) may be an artifact of the 2‐min sampling rate, whereby individuals traveled beyond the recorded maximum depth and began ascending between consecutive records, resulting in a rounded dive apex (Wilson et al. 1995). Therefore, linear descents may represent a more prevalent shape than our analysis indicated. Descent cluster designation did not appear to be biologically meaningful as environmental variables and dive characteristics did not vary markedly between clusters. In contrast, ascent shape related to all considered environmental variables. Closer inspection of the systematic and frequent transition point ascents (TPAs) and linear ascents (LAs) revealed that the characteristics of these clusters are closely related to the ambient environment, particularly conditions at dive apex.

### Functionality of mesopelagic excursions

Hypotheses for the function of MEs by epipelagic sharks include interactive behaviors (reproduction, predator avoidance, etc.), energy conservation, behavioral thermoregulation, navigation, and foraging (Carey and Scharold 1990; Campana et al. 2011; Gleiss et al. 2011; Jorgensen et al. 2012). Here, we discuss some of the predictions of each hypothesis in the context of the results obtained for oceanic whitetip sharks.

#### Interactive behaviors

All tracked sharks were mature, raising the possibility that MEs had a reproductive function, such as courtship or mating. However, in other species such as lamnid sharks, mating occurs in specific locations and seasons (Conrath and Musick 2012), as observed by site‐specific deep‐diving lekking displays demonstrated by white sharks in the Pacific Ocean (Jorgensen et al. 2012). In contrast, MEs by oceanic whitetips occurred throughout the year, near the aggregation site, and throughout the migratory circuit (presumably when solitary). With the exception of one individual tracked for 22 days, all sharks in this study exhibited MEs. Moreover, oceanic whitetips have a suggested biennial reproductive cycle (Tambourgi et al. 2013) so that only some females will mate in a given mating season (the others will be pupping). Anecdotal and recorded observations report oceanic whitetips interacting with recreational and commercial fishing vessels, but besides anthropogenic activity, it is unlikely that this large‐bodied predator (with an estimated 4.2 trophic level) regularly encounters other predators (Backus et al. 1956; Howey‐Jordan et al. 2013; Madigan et al. 2015). Consequently, we do not consider interactive behaviors to be a viable hypothesis.

#### Energy conservation

Deep diving may be a mechanism for energy conservation by allowing the negatively buoyant animal to rest on gliding descents (Weihs 1973). While studies have modeled energy conservation methods by examining diving dynamics and power consumption (Gleiss et al. 2011; Iosilevskii et al. 2012), application of such theories requires more information than one‐dimensional vertical projection of movement. However, considering the relative duration of dive phases, Meekan et al. (2015) showed that energy savings in whale shark dives were the result of the long‐duration descents followed by shorter duration ascents. In contrast, MEs in oceanic whitetips exhibited the opposite pattern and were characterized by relatively short descents followed by longer duration ascents. Therefore, we suspect any energy conserved on the short‐duration descent may be drained in the following high‐energy ascent. Additionally, in a long‐distance migrant species such as the oceanic whitetip shark, we postulate that if MEs reduced the cost of horizontal transport, they would be more frequently observed during time intervals associated with large displacements. Dives occurred at the aggregation site and during migration, which is inconsistent with the energy conservation hypothesis.

#### Behavioral thermoregulation

The behavioral thermoregulation hypothesis suggests individuals may dive below the thermocline to dissipate excess heat absorbed from surface waters (Thums et al. 2012). Although oceanic whitetips demonstrated increasing dive frequency with warming SST, examination of pre‐diving tag temperature revealed that mean temperatures experienced prior to MEs were actually cooler than randomly selected nondiving periods. Therefore, no evidence exists to suggest MEs were driven by heat acquired in warm surface waters. Additionally, while studies investigating thermal inertia in oceanic whitetip sharks do not exist, the duration of mesopelagic excursions (<65 min) was shorter than would likely be required to significantly lower body temperature. A temperature change of 3°C in leopard sharks (*Triakis semifasciata*) took approximately one hour (Hight and Lowe 2007). As a larger bodied species, we suspect oceanic whitetip sharks exhibit greater thermal inertia than leopard sharks. Furthermore, the hysteresis of body temperature variation during deep‐water dives likely offsets any persisting cooling effect as studies report that blue shark body temperature decreases slower on descents than ascents, suggesting any heat lost on the rapid descent may be quickly recovered along the ascent (Carey and Gibson 1987; Carey and Scharold 1990). While we do not discount possible physiological temperature effects during MEs, we believe it is unlikely that thermoregulation is the primary function of these dives.

#### Navigation

The navigation hypothesis suggests that oceanic whitetips perform MEs to obtain bathymetric or geomagnetic cues to aid in navigation such as the dives exhibited by scalloped hammerheads, reaching depths ≥980 m and lasting approximately 30 min (Klimley 1993; Klimley et al. 2002; Jorgensen et al. 2009). Deep dives associated with magnetic reception in southern bluefin tuna demonstrated predictable shapes during dawn and dusk periods, as these are the times when magnetic gradients are likely to be strongest (Willis et al. 2009). Similarly, oceanic whitetip MEs were consistently shaped, directed excursions, occurring most frequently during dusk; no significant pattern during the dawn period was identified. To date, TPAs have not been documented in other well‐studied, highly migratory, epipelagic shark species (e.g., white sharks, blue sharks, shortfin mako sharks [*Isurus oxyrinchus*], tiger sharks [*Galeocerdo cuvier*]). The physiological mechanism(s) used to navigate an expansive oceanic environment, while poorly understood in pelagic sharks, is unlikely to vary among species. MEs may also serve as a simple way to ascertain seafloor depth. Individuals may be able to gain locational insight by attempting to “ground truth” seafloor depth and use this information to construct generalizations about location relative to islands and land masses. The low frequency of TPAs and the highly migratory nature of oceanic whitetip sharks suggest navigation is an unlikely motivator for MEs. However, the poorly understood mechanism(s) of navigation in oceanic vertebrates precludes this hypothesis from being discounted.

#### Foraging

Several studies have associated diving behavior with foraging in sharks (Carey and Scharold 1990; Domeier and Nasby‐Lucas 2008; Nasby‐Lucas et al. 2009; Carlisle et al. 2012; Jorgensen et al. 2012). A recent study showed that biomass of deepwater mesopelagic fishes could be 10 times higher than historically believed (Irigoien et al. 2014), and the abundance of deepwater organisms may provide a food source for species usually associated with epipelagic zone (Sutton 2013). White sharks aggregate in a highly productive area in the central North Pacific where they change their surface‐oriented behavior associated with migration to one punctuated by rapid dives between the surface and depths up to 500 m (Weng et al. 2007; Nasby‐Lucas et al. 2009; Jorgensen et al. 2012). These rapid oscillatory dives have been linked to foraging in the deep scattering layer (Carlisle et al. 2012; Jorgensen et al. 2012).

Stable isotope and stomach content analyses indicate that oceanic whitetip diets consist of epipelagic predatory teleosts, forage fishes, and squid (Cortes 1999; Madigan et al. 2015). Analysis of muscle tissue sample collected from oceanic whitetip sharks at the Cat Island aggregation, where sharks were tagged for the present study, estimated that pelagic squid constituted 44% of oceanic whitetip sharks' long‐term (>1 year) diet while short‐term (70–200 day) diet consisted of 23% squid (Madigan et al. 2015). Although the species of squid could not be identified in the analysis, many species of squid inhabit Atlantic waters below the thermocline (Voss and Brakoniecki 1985). Other species, such as swordfish (*Xiphias gladius*) and shortfin mako sharks, also consume squids in the mesopelagic zone (Toll and Hess 1981; Stillwell and Kohler 1982; Loefer et al. 2005; Vetter et al. 2008; Wood et al. 2009). Furthermore, a greater number of MEs were recorded during dusk periods when many sharks are known to increase activity (e.g., Lowe 2002; Gleiss et al. 2013). Many species of meso‐ and bathypelagic squids undergo diel vertical migrations, with upward migrations occurring at dusk (Roper and Young 1975). Thus, the dusk period may offer oceanic whitetip sharks an advantage during pursuit of their migrating prey. Even during nondusk diel periods, many cephalopod species exhibit vertical distributions that overlap the depth of MEs (Vecchione and Roper 1991). Additionally, oceanic whitetip sharks are known to associate with short‐finned pilot whales (*Globicephala macrorhynchus)* (Stafford‐Deitsch 1988). Aguilar Soto et al. (2008) reported short‐finned pilot whales in the eastern North Atlantic make foraging dives reaching 1019 m, lasting up to 21 min, before resurfacing. These depths and durations are comparable to MEs in oceanic whitetip sharks, suggesting these species may be exploiting similar prey resources.

Fast and directed descents, like those observed in the Descent 1 cluster, may be further indicators of foraging behavior. In an examination of whale shark diving, Thums et al. (2012) identified a “type 3” dive, presumably for feeding. Similar to oceanic whitetip dive profiles, these dives also exhibited descents with faster vertical velocities than corresponding ascents. Dive timing, prey constituents, and dive shape support foraging as a viable motive for MEs.

### Behavioral variations in mesopelagic excursion ascents

Transition point ascents and LAs differed by the fast initial segment (Segment 1) characteristic of TPAs, but the second segment (Segment 2) of the TPA is indistinguishable from the LAs. The reason why individuals either ascended from a dive at an initially faster vertical velocity (TPAs) or a comparatively slower vertical velocity (LAs) remains unknown. At the transition point, the rapid decrease in vertical velocity may be the result of decreased swimming rate, inclination (shallowing of the animal's pitch angle), or both. Another possibility to explain the decreased ascent rate during TPAs is a change from (near‐) straight‐line swimming to a sinuous (side‐to‐side) swimming pattern. Regardless of the behavior change at the transition point, LAs would likely offer a more energetically efficient means to return to surface waters (Nakamura et al. 2011). Thus, we postulate the additional debt incurred during the initial period of TPAs (Segment 1) is offset by procurement of advantageous resources (or avoidance of further debt) present during that phase of the ascent. We suggest the different ascent modes represent distinct responses to factors, either environmental or related to prey distribution, experienced at the dive apex.

#### Environmental response

Variations in environmental parameters related to ascent characteristics within each ascent mode. For both LAs and TPAs, the greater depths and colder temperatures encountered resulted in faster vertical velocities during the ascent. Furthermore, depths and temperatures at the transition point were closely related to the environment at the dive apex. Deeper (and colder) dives exhibited correspondingly deeper (and colder) transition points and faster vertical velocities. These ascent characteristics may suggest that sharks incur some debt from diving deeper, perhaps related to intolerable temperatures, oxygen levels or pressure ratios. However, given the collinearity between abiotic variables, it is a challenge to discern which variable(s), if any, fundamentally influence the vertical velocity of ascents.

Temperature is an important factor in determining horizontal and vertical habitat use in ectothermic sharks (Block et al. 2011). Blue sharks, also ectotherms, more commonly associate with temperate waters but migrate into tropical latitudes during winter months while demonstrating tropical submergence (Strasburg 1958; Howey 2010; Campana et al. 2011). In contrast, the distribution of ectothermic oceanic whitetip sharks in the western North Atlantic appears limited to warm surface waters in tropical and subtropical latitudes and water masses that warm in summer months (e.g., Gulf of Mexico and Gulf Stream) (Bonfil et al. 2008; Castro 2011; Howey‐Jordan et al. 2013; Froese and Pauly 2015). However, it remains unclear whether this range reflects physiological limitations in thermal tolerance or prey distribution, as prey also have unknown thermal requirements.

Several shark species exhibit vertical distributions limited by oxygen levels, with only occasional excursions into the potentially hypoxic habitat (Vetter et al. 2008; Jorgensen et al. 2009; Nasby‐Lucas et al. 2009; Gilly et al. 2013). As a response to hypoxia, ectothermic obligate ram‐ventilators increase swimming speed (and mouth gape) (Carlson and Parsons 2001). Correspondingly, the possibility exists that oceanic whitetip sharks were responding to hypoxic conditions during ME ascents, traveling faster (TPA Segment 1) through hypoxic regions. Yet, our study identified TPAs that did not enter the OMZ and LAs that entered the OMZ. While we acknowledge that error may be amplified when tag‐estimated light‐based geolocations and the dissolved oxygen grid (Garcia et al. 2013) are overlaid, further research is required in order to understand the oceanic whitetips' response to hypoxic conditions.

Although TPAs were generally associated with colder and deeper MEs, considerable overlap existed between TPAs and LAs in the water column, with the deepest LA occurring from a dive apex of 751 m (10.89°C). Therefore, while the possibility exists that the ascent mode may be a response to ambient conditions, the overlap of TPAs and LAs suggests otherwise.

#### Foraging behavior

Given the overlap of TPAs and LAs in the water column, we suspect another factor(s) may be influencing the ascent characteristics. Furthermore, as foraging appears to be the most viable explanation for MEs, we must assess the ascent shape as linked to foraging behavior. As previously mentioned, the diets of oceanic whitetips include squid (species unknown) (Madigan et al. 2015). Many squids undergo diel vertical migrations and concentrate in species‐specific depth bands in waters below the thermocline (Roper and Young 1975; Voss and Brakoniecki 1985).

Oceanic whitetips may dive to aphotic depths to locate potential prey using a variety of sensory mechanisms (e.g., vision, olfaction, electroreception) (Gardiner et al. 2012). If potential prey are found, the sharks may attempt to feed. To engage prey items, sharks could ascend quickly through the prey patch, corresponding to the initial fast ascent Segment 1 of TPAs. Upon exiting the prey band, presumably after consuming prey, sharks may resume an energetically efficient ascent mode by adjusting pitch and/or speed, represented by the consistent Segment 2 in TPAs. In contrast, LAs may be indicative of a dive in which no prey were detected. For example, if a shark fails to locate prey during the descent, the shark may ascend in the efficient manner represented in LAs. As additional support for this theory, a foraging study indicates that predators modify search patterns as a response to prey availability and abundance (Humphries et al. 2010).

While we identified a relationship between ascent mode and ambient conditions, this result could be confounded by the abiotic habitat requirements of the prey resource. Further diet studies could help determine whether the ME characteristics were related to the habitat use of prey.

## Conclusions

Meso‐ and bathypelagic excursions are regularly exhibited by a variety of fishes generally associated with epipelagic waters (Sutton 2013). Despite spending a minute portion of their life history at aphotic depths, use of these habitats in oceanic whitetips occurred throughout the year, during migration and near the aggregation in The Bahamas. The widespread frequency at which MEs occurred, and the presumed energetic cost, suggests that use of aphotic habitats is biologically significant and provides a benefit. Of the hypotheses suggested to explain the function of oceanic whitetip MEs, only navigation and foraging were considered viable. Although poorly understood in sharks, the navigational mechanisms used in migration are unlikely to vary among species. In contrast, while most shark species are likely opportunistic with regard to prey, most sharks likely exhibit species‐specific diets. Given that TPAs have not been identified in other epipelagic shark species, and based on available data, we suggest MEs are more likely to represent foraging behavior than a mechanism for navigation. However, a study specifically designed to identify foraging events during MEs is required to confirm our proposed theory of dive functionality. Further understanding of navigational mechanisms is also needed before this theory can be discounted. Biologically relevant dive patterns were largely the result of variations in the ascent phase of the MEs. It appears LAs and TPAs represent a different behavioral response to conditions at the dive apex. While ascent shape clearly associated with the ambient environment, overlap of LAs and TPAs in the water column and the hypothesized foraging function suggest that ascent profile shape may represent intentional behavior, such as interactions with prey.

## Data Accessibility

Mesopelagic excursions in oceanic whitetip sharks. KNB Data Repository. doi:10.5063/F1SJ1HHD.

## Conflict of Interest

Three of the authors, Lucy A. Howey, Emily R. Tolentino, and Lance K.B. Jordan, are currently employed by the manufacturer of satellite tags used in this study, which had no bearing on study design, implementation, or results.

## Supporting information


**Table S1.** Summary of biological and X‐Tag information from 16 tagged sharks.
**Data S1.** Sampling rate analysis.
**Figure S1.** Six‐hour data subsample from accelerometer depth data logger.
**Data S2.** Data treatment.
**Data S3.** Generalized linear mixed model details.
**Table S2.** Summary of negative binomial models.
**Table S3.** Summary of linear mixed models.
**Data S4.** Supporting information references.
