# Supplementary material for: Into the deep: the functionality of mesopelagic excursions by an oceanic apex predator
Source: Ecol Evol. 2016 Jun 28;6(15):5290–304. doi: 10.1002/ece3.2260 (PMC4984504; doi:10.1002/ece3.2260)
Supplement: Supplementary file 1 — Table S1. Summary of biological and X‐Tag information from 16 tagged sharks. Data S1. Sampling rate analysis. Figure S1. Six‐hour data subsample from accelerometer depth data logger. Data S2. Data treatment. Data S3. Generalized linear mixed model details. Table S2. Summary of negative binomial models. Table S3. Summary of linear mixed models. Data S4. Supporting information references. [file ECE3-6-5290-s001.pdf]

SUPPORTING INFORMATION

to

Into the deep: the functionality of mesopelagic excursions by an oceanic apex predator

Lucy A. Howey[a], Emily R. Tolentino[a], Yannis P. Papastamatiou[b], Edward J. Brooks[c], Debra L. Abercrombie[d], Yuuki Y. Watanabe[e,f], Sean Williams[c], Annabelle Brooks[c], Demian D. Chapman[g], and Lance K.B. Jordan[a*]

[a] Microwave Telemetry, Inc., Columbia, Maryland, USA

[b] Department of Biological Sciences, Florida International University, North Miami, Florida, USA

[c] Shark Research and Conservation Program, Cape Eleuthera Institute, The Bahamas

[d] Abercrombie & Fish, Miller Place, New York, USA

[e] National Institute of Polar Research, Tachikawa, Tokyo, Japan

[f] Department of Polar Science, SOKENDAI (The Graduate University for Advanced Studies), Tachikawa, Tokyo, Japan

[g] School of Marine and Atmospheric Science & Institute for Ocean Conservation Science, Stony Brook University, Stony Brook, New York, USA

*Corresponding author: jordanl@nova.edu

Table S1. Summary of biological and X-Tag information from 16 tagged sharks.

X-Tag Argos ID	Deployment date	Days at liberty	Programmed deployment duration (months)	Fork length (cm)	Sex	Pop-off latitude (°N)	Pop-off longitude (°W)	Mean (± SD) depth (m)	Mean (± SD) temperature (°C)	Maximum depth (m)	Minimum temperature (°C)
107797	5/1/2011	184	6	218	F	22.89	74.68	59.92 ± 45.02	26.35 ± 1.68	1081.9	7.75
107798	5/4/2011	153	5	189	F	23.20	74.91	46.02 ± 38.52	26.16 ± 1.87	1008.7	8.54
107799	5/4/2011	245	8	214	F	23.73	74.93	50.03 ± 37.29	26.59 ± 1.46	751.8	10.89
107805	5/1/2011	153	5	233	F	24.36	75.37	41.66 ± 37.42	25.98 ± 1.66	720.2	7.90
119869	5/7/2012	245	8	212	F	24.60	75.50	43.32 ± 33.9	26.18 ± 1.56	577.3	13.53
119870	5/7/2012	150	8	179	F	22.69	73.38	50.8 ± 39.9	27.09 ± 1.65	635.1	11.04
119872	5/11/2012	278	11	228	F	23.94	74.93	45.37 ± 48.83	26.34 ± 1.74	1190.2	7.43
119874	5/12/2012	335	11	212	F	24.75	75.52	45.82 ± 40.55	25.29 ± 1.54	1081.9	6.79
119878	5/12/2012	325	11	195	M	24.01	74.46	57.65 ± 41.11	25.33 ± 1.56	875.2	9.48
119882	5/9/2012	112	11	216	F	24.22	75.32	74.94 ± 44.19	26.58 ± 1.70	845.6	9.32
119891	5/10/2012	60	8	168	F	24.67	76.05	37.28 ± 29.26	26.04 ± 1.26	420.6	17.63
119893	5/9/2012	245	8	210	F	21.77	74.20	48.64 ± 43.02	26.31 ± 1.65	895.3	9.48
129911	5/8/2013	83	12	198	M	23.11	75.02	42.04 ± 41.29	25.95 ± 1.32	855.3	9.63
129922	5/9/2013	174	10	183	F	25.30	76.34	46.63 ± 33.04	26.36 ± 1.56	442.1	17.94
129925	5/12/2013	202	10	203	F	24.63	75.83	45.04 ± 35.54	26.56 ± 1.53	838.5	9.32
129933	5/8/2013	22	8	203	F	24.83	75.70	39.42 ± 30.49	25.33 ± 0.94	232.0	20.21

Data S1. SAMPLING RATE ANALYSIS

A preliminary analysis was conducted to investigate if the recovered X-Tag 2-minute temporal resolution was adequate for capturing vertical movements of oceanic whitetip sharks. This analysis required oceanic whitetip depth data believed to capture all significant vertical movements. As part of an ongoing study into fine-scale swimming mechanics of oceanic whitetip sharks, we attached activity sensing data loggers and accelerometers, which incidentally provided the high-resolution data needed to assess the adequacy of the recovered X-Tag temporal resolution.

A float and PD3GT data logger (Little Leonardo, Tokyo, Japan) package was deployed on a mature female oceanic whitetip (FL = 216 cm) on May 8, 2013 near Cat Island, The Bahamas (24.108 N, 75.274 W). The package was equipped with a 3D accelerometer (sampling rate 16 Hz), as well as depth (resolution 0.25 m) and temperature loggers providing data on 1-second intervals. The data logger package was inserted into a float attached via nylon cable tie through two small holes made in the dorsal fin. After the three day deployment period, the time-release mechanism jettisoned the entire package from the animal, allowing it to float to the surface. Attached VHF and SPOT (Wildlife Computers, Redmond, WA, USA) transmitters enabled the physical recovery of the package and subsequent data retrieval.

A total of 315,555 depth records was obtained from the data logger package after being deployed for 3.7 days. First, a 2-minute depth subsample was extracted and visually compared to the 1-second profile (Fig. S1), indicating that the 2-minute subsample captures most vertical movements. Next, the 2-minute subsample was linearly interpolated on 1-second intervals. The mean absolute difference between corresponding profile points of the linearly interpolated 2-minute profile and the 1-second profile was less than 1 m (0.924 ± 1.826 m). Furthermore, approximately one third (32.5%) of the linearly interpolated records were within ±0.25 m of the corresponding 1-second profile points. Lastly, each 2-minute section (n = 2651) of the 1-second profile was considered. The difference between the first and last records in each 2-minute interval defined a depth range. Within each 2-minute section, the vertical excursions beyond (above and below) the depth range were summed, providing an estimate of the vertical movements not captured by the 2-minute sampling rate. The resulting mean magnitude of depth excursions not captured by consecutive 2-minute records was 0.877 ± 1.416 m and ranged from 0 to 28.75 m. Over a quarter of the intervals (28.6%) did not register any depth excursions beyond the 2-minute depth band. Fifty-four intervals (2.04%) failed to capture >5 m of vertical movement, and only 4 intervals (0.15%) failed to capture >10 m of movement.

Results demonstrate that recovered X-Tag depth-versus-time profiles capture the general vertical behavior of oceanic whitetip sharks.

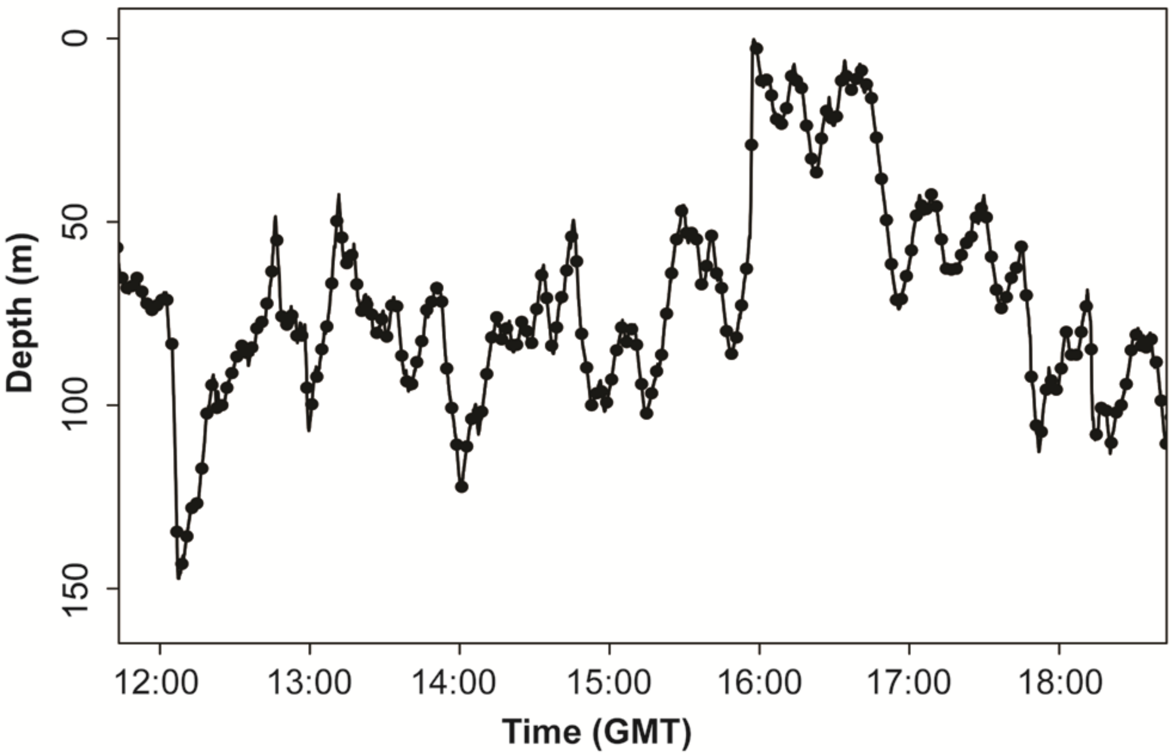

Figure S1. Six-hour data subsample from accelerometer depth data logger. Solid line represents 1-second depth profile, and points represent 2-minute subsample.

Data S2. DATA TREATMENT

All depth and temperature records from the day of deployment and detachment were removed from the analyzed dataset.

In order to estimate the location of mesopelagic excursions (MEs), daily light-based geolocations from X-Tags were processed with a state-space unscented Kalman filter with sea surface temperature (UKFSST) (Reynolds et al. 2002; Lam et al. 2008; http://www.esrl.noaa.gov/psd/*)* and a subsequent bathymetric correction (Galuardi et al. 2010). Since individuals spent considerable time near the surface, daily sea surface temperature (SST) estimates were based on daily maximum temperature records provided by the X-Tags (Galuardi and Lutcavage 2012). The 'analyzepsat' package in R was used for location processing (Galuardi 2012; Galuardi and Lutcavage 2012).

Each ME was assigned a location based on the day's corresponding UKFSST estimate. In addition, a diel period (dawn, day, dusk, or night) was allocated to each ME based on the time-stamp of the first dive record, specifying dawn and dusk as the two-hour periods centered on the UKFSST-derived sunrise and sunset times. Similarly, each excursion was assigned to one of four categorical lunar phases: first quarter, full moon, last quarter, and new moon (http://aa.usno.navy.mil/index.php). These lunar phase periods

were defined as the approximately weeklong time periods centered on the date of the first quarter, full, last quarter, and new moon phases.

Since X-Tags record depth and temperature concurrently, ME depth profiles were annotated with tag-recorded temperature. Additionally, dive profiles were annotated with vertically interpolated (cubic spline) objectively analyzed annual mean dissolved oxygen (mL/L) retrieved from NOAA NODC World Ocean Atlas 2013 (Garcia et al. 2013). Oxygen data had a spatial resolution of 1° (Latitude/Longitude) and were geographically aligned with dive locations by identifying the closest grid cell with data covering the corresponding ME depth range.

Data S3. GENERALIZED LINEAR MIXED MODEL DETAILS

Three negative binomial models were constructed to assess the significance of factors predicting mesopelagic excursion (ME) events (Table S2).

In the model evaluating ME frequency in relation to SST, individual monthly dive frequency (counts) was modeled as the response variable dependent on the continuous monthly mean SST covariate. Monthly mean SST was estimated by monthly mean daily maximum recorded temperature for each individual. Therefore, mean SST was determined for each individual independently. The temporal offset term was the number of tracking days per month for each individual. The model included the mean SST covariate as both a fixed effect and random effect within each individual (Table S2). Determination of the random effect structure was based on comparison of Akaike information criterion (AIC) values from candidate models.

In the model for ME frequency with respect to the diel period factor (dawn, day, dusk, and night), each individual contributed four counts (responses), one for each diel period. The temporal offset term was the total tracking hours in each diel period determined for each individual (Table S2).

In the last negative binomial model considering ME frequency with respect to the discrete lunar phase factor (first quarter, full, last quarter, and new moon), ME events were counted in each lunar phase for each individual. Therefore, each individual contributed four counts (responses) to the model. The temporal offset was the number of total tracking days each individual spent in each lunar phase (Table S2).

Table S2. Summary of negative binomial models.

Response variable	Temporal offset (log-transformed)	Covariate	Fixed effect levels	Random effect
Monthly ME frequency	Number tracking days per month for each individual	Monthly mean SST	Continuous	SST within individual
ME frequency per diel period	Total tracking hours in diel period for each individual	Diel period	Dawn, day, dusk, night	Individual
ME frequency per lunar phase	Total tracking days in lunar phase for each individual	Lunar phase	First quarter, full, last quarter, new	Individual

A series of linear mixed effect (LME) models was constructed to investigate various relationships (Table S3). In order to rectify violations of linear model assumptions, a heteroscedastic variance structure was incorporated in models exhibiting unequal variances between groups, and an autoregressive moving average (ARMA) correlation structure (with autoregressive order *p* and moving average order *q*) was introduced in models to account for correlated residuals between ordered observations within each individual (Pinheiro and Bates 2000) (Table S3).

In the model predicting mean temperature before mesopelagic excursions versus randomly selected periods spent strictly in the epipelagic zone, various time periods were considered (30 min, 1–5 h). Therefore, the model was evaluated 6 times, each time considering the mean temperature response variable over different durations.

Dive maximum depth (m) and dive minimum temperature (°C) were identified as the deepest and coldest records in the dive, respectively. Mean ascent vertical velocity (m/s) and descent vertical velocity (m/s) were calculated by dividing descent and ascent vertical displacements by the time duration of the respective phase profiles. For each descent, the corresponding mean ascent vertical velocity was calculated. Similarly, for each ascent, the corresponding descent vertical velocity was calculated. Therefore, the mean vertical speeds before and after the ascent and descent clusters could be considered. The minimum dissolved oxygen (mL/L) value was the lowest oxygen concentration experienced during the dive. Lastly, the duration (min) was identified as the time duration of the individual ascent or descent phase profiles.

Table S3. Summary of linear mixed models.

Response variable	Fixed effect	Fixed effect levels	Random effect	ARMA	Variance structure
Mean temperature (°C)	Period type	Pre-ME, Non-ME	Period type within individual	*p* = 1, *q* = 1	-
Dive maximum depth* (m)	Ascent Cluster + Descent Cluster	Ascent Cluster: Ascent 1–3 Descent Cluster: Descent 1–3	Individual	-	-
Dive minimum temperature* (°C)	Ascent Cluster + Descent Cluster	Ascent Cluster: Ascent 1–3 Descent Cluster: Descent 1–3	Individual	-	-
Mean descent vertical velocity* (m/s)	Ascent Cluster + Descent Cluster	Ascent Cluster: Ascent 1–3 Descent Cluster: Descent 1–3	Individual	-	-
Mean ascent vertical velocity* (m/s)	Ascent Cluster + Descent Cluster	Ascent Cluster: Ascent 1–3 Descent Cluster: Descent 1–3	Individual	*p* = 1, *q* = 1	Within individual
Dive minimum dissolved oxygen* (mL/L)	Ascent Cluster + Descent Cluster	Ascent Cluster: Ascent 1–3 Descent Cluster: Descent 1–3	Individual	*p* = 1, *q* = 0	Within ascent cluster
Duration* (min)	Cluster	Ascent 1–3, Descent 1–3	Individual	-	Within cluster
Mean vertical velocity* (m/s)	Cluster Segment	Ascent 1, Ascent 2 Segment 1, Ascent 2 Segment 2	Individual	*p* = 1, *q* = 1	Within cluster segment

* Indicates Box-Cox transformation

Data S4. SUPPORTING INFORMATION REFERENCES

Galuardi, B. 2012. analyzepsat: Microwave Telemetry PSAT analysis. R package version 3.1.

Galuardi, B., and M. Lutcavage. 2012. Dispersal routes and habitat utilization of juvenile Atlantic bluefin tuna, *Thunnus thynnus*, tracked with mini PSAT and archival tags. PLoS ONE 7, e37829.

Galuardi, B., F. Royer, W. Golet, J. Logan, J. Neilson, and M. Lutcavage. 2010. Complex migration routes of Atlantic bluefin tuna (*Thunnus thynnus*) question current population structure paradigm. Can. J. Fish. Aquat. Sci. 67:966–976.

Garcia, H. E., R. A. Locarnini, T. P. Boyer, J. I. Antonov, A. V. Mishonov, O. K. Baranova, et al. 2013. World Ocean Atlas 2013. Volume 3: Dissolved oxygen, apparent oxygen utilization, and oxygen saturation *in* S. Levitus, A. Mishonov, eds.. NOAA Atlas NESDIS 75.

Lam, C.H., A. Nielsen, and J. R. Sibert. 2008. Improving light and temperature based geolocation by unscented Kalman filtering. Fish. Res. 91:15–25.

Pinheiro, J. C., and D. M. Bates. 2000. Mixed-effects Models in S and S-Plus. Springer-Verlag, New York.

Reynolds, R.W., N. A. Rayner, T. M. Smith, D. C. Stokes, and W. Wang. 2002. An improved in situ and satellite SST analysis for climate. J. Climate 15:1609–1625.
